# Critical Care Management of Severe Acute Pancreatitis: Current Concepts, Clinical Challenges, and Future Perspectives

**DOI:** 10.3390/life16091407

**Published:** 2026-08-25

**Authors:** Sándor Márton

**Affiliations:** Department of Anaesthesiology and Intensive Therapy, University of Pécs Medical School, 7624 Pécs, Hungary; marton.sandor@pte.hu

**Keywords:** acute pancreatitis, severe acute pancreatitis, intensive care, organ failure, fluid resuscitation, pancreatic necrosis, step-up approach, enteral nutrition, endoscopy, critical care

## Abstract

Acute pancreatitis is a common and heterogeneous inflammatory disorder whose clinical course ranges from a self-limited illness to persistent organ failure, infected pancreatic necrosis, and prolonged critical illness. Contemporary management has moved away from protocolised aggressive fluid loading, prolonged fasting, prophylactic antibiotics, and early open necrosectomy. Instead, current care emphasises repeated physiological assessment, moderate goal-directed resuscitation, early enteral or oral nutrition, organ-specific support, antimicrobial stewardship, and delayed minimally invasive intervention within a multidisciplinary step-up strategy. This narrative review examines acute pancreatitis from an intensive care perspective. Particular attention is given to early risk stratification, intensive care unit triage, haemodynamic and respiratory support, acute kidney injury, intra-abdominal hypertension, nutrition, biliary source control, diagnosis and treatment of infected necrosis, and the timing and selection of endoscopic, radiological, and surgical interventions. The implications of obesity, pregnancy, advanced age, and multimorbidity are also discussed. Recent randomised trials have clarified several clinically important questions: aggressive hydration increases fluid overload without improving outcomes; routine urgent endoscopic retrograde cholangiopancreatography is not beneficial in predicted severe biliary pancreatitis without cholangitis; postponed drainage may avoid invasive intervention in a substantial proportion of patients with infected necrosis; and endoscopic or minimally invasive approaches reduce treatment burden compared with primary open surgery. Persistent organ failure remains the principal determinant of mortality, while infected necrosis further increases risk and complexity. Future progress will depend on dynamic prediction models, biomarker-guided antimicrobial decisions, personalised haemodynamic strategies, phenotype-directed immunomodulation, and regionalised multidisciplinary care.

## 1. Introduction

Acute pancreatitis (AP) is among the most frequent gastrointestinal causes of acute hospital admission, and its incidence is increasing in many regions. Most patients experience interstitial oedematous pancreatitis and recover with supportive treatment; however, a clinically important minority develop necrosis, persistent organ failure, infected collections, or a combination of these complications [1,2,3,4,5,6,7,8,9,10]. Severe AP is not simply a local pancreatic disorder. It is a systemic critical illness characterised by endothelial dysfunction, capillary leak, microcirculatory impairment, dysregulated innate immunity, and progressive respiratory, cardiovascular, and renal dysfunction.

The management paradigm has changed substantially during the past two decades. Early open necrosectomy, routine prophylactic antibiotics, prolonged pancreatic ‘rest’, and indiscriminate high-volume crystalloid therapy have been replaced by less invasive, physiology-driven strategies [2,3,4,5,6,11,12,13,14,15]. The 2025 international guideline update and the 2024 American College of Gastroenterology guideline consolidate this shift and emphasise dynamic reassessment, moderate rather than aggressive fluid therapy, early nutrition, avoidance of unnecessary ERCP, and minimally invasive intervention for necrotising disease [2,3].

This review focuses on the decisions most relevant to anaesthesiologists and intensivists: recognising patients at risk of deterioration, selecting the appropriate level of monitoring, supporting failing organs without amplifying iatrogenic injury, distinguishing sterile inflammation from infection, and coordinating the timing of endoscopic, radiological, and surgical source control.

## 2. Literature Search Strategy

A clinically oriented narrative review was performed. PubMed/MEDLINE and the websites of major professional societies were searched for English-language publications published from January 2000 to July 2026. Search terms were used in combinations and included ‘acute pancreatitis’, ‘severe acute pancreatitis’, ‘critical care’, ‘intensive care’, ‘organ failure’, ‘fluid resuscitation’, ‘nutrition’, ‘infected necrosis’, ‘endoscopic drainage’, ‘necrosectomy’, ‘abdominal compartment syndrome’, ‘risk prediction’, ‘obesity’, ‘pregnancy’, and ‘machine learning’. Eligible sources comprised international or national guidelines, randomised controlled trials, prospective multicentre studies, systematic reviews, meta-analyses, and clinically influential observational studies addressing diagnosis, severity assessment, organ support, nutrition, infection, or intervention in acute pancreatitis. Case reports, small uncontrolled series without practice-relevant information, non-English publications, conference abstracts without a full report, and articles not directly relevant to critical care decision-making were excluded. Landmark publications predating 2000 were retained when they established definitions, prognostic systems, or interventional strategies still used in current practice. Titles and abstracts were screened for clinical relevance, after which full texts of potentially eligible publications were assessed; reference lists of major guidelines and reviews were also examined to identify additional landmark studies. Evidence was selected purposively according to methodological quality, clinical relevance, and its contribution to contemporary management. Because this was a narrative rather than systematic review, a prospectively documented screening log was not maintained; consequently, retrospective counts of screened and excluded records cannot be reported reliably. No formal risk-of-bias assessment or quantitative meta-analysis was undertaken.

## 3. Diagnosis, Morphology, and Severity Classification

The diagnosis of AP requires at least two of three features: characteristic upper abdominal pain, serum amylase or lipase greater than three times the upper limit of normal, and imaging findings consistent with AP [1,2,3,4]. Serum lipase is generally preferred. Early cross-sectional imaging is unnecessary when the clinical and biochemical diagnosis is secure, because necrosis may be underestimated during the first days and imaging rarely changes management in uncomplicated disease. Contrast-enhanced computed tomography (CT) or magnetic resonance imaging is most useful when the diagnosis is uncertain, deterioration occurs, complications are suspected, or intervention is being planned [2,3,4,5].

The revised Atlanta classification separates interstitial oedematous pancreatitis from necrotising pancreatitis and categorises severity as mild, moderately severe, or severe [1]. Severe AP is defined by persistent organ failure lasting more than 48 h, usually quantified with the modified Marshall score. This definition is clinically important because persistent organ failure, rather than the anatomical extent of pancreatic inflammation alone, is the dominant determinant of early mortality [1,16,17]. Local collections are classified according to content and time: acute peripancreatic fluid collection, pancreatic pseudocyst, acute necrotic collection, and walled-off necrosis. Precise terminology prevents premature or inappropriate intervention.

CT-based scores, including the Balthazar CT Severity Index and the modified CT Severity Index, describe inflammation, necrosis, and extrapancreatic complications [18,19]. They complement, but do not replace, repeated bedside assessment. Imaging severity and physiological severity may diverge; a patient can have extensive necrosis without current organ failure or profound organ failure before necrosis is fully apparent.

## 4. Pathophysiological Basis of Critical Illness

Acinar cell injury, premature intracellular trypsin activation, disturbed calcium signalling, mitochondrial dysfunction, and impaired autophagy initiate pancreatic inflammation. Damage-associated molecular patterns activate macrophages, neutrophils, complement, and cytokine networks, producing local oedema and systemic endothelial injury. Microvascular leakage reduces effective circulating volume while simultaneously promoting pulmonary and tissue oedema. The resulting combination of hypovolaemia, vasoplegia, myocardial depression, and microcirculatory dysfunction explains why simple replacement of a presumed ‘third-space deficit’ with large fixed fluid volumes is physiologically inadequate and potentially harmful [4,5,6,11,20].

Persistent systemic inflammatory response syndrome (SIRS) is associated with organ failure and adverse outcomes [21]. In later phases, compensatory immunosuppression may coexist with continuing inflammation, increasing vulnerability to bacterial translocation, infected necrosis, catheter-related infection, pneumonia, and fungal infection. This temporal overlap complicates interpretation of fever, leucocytosis, and C-reactive protein. Treatment therefore requires serial evaluation of the entire clinical trajectory rather than reliance on a single laboratory threshold.

## 5. Early Risk Stratification and ICU Triage

No single score reliably predicts the course of AP at presentation. Useful early warning features include persistent SIRS, rising blood urea nitrogen (BUN), haemoconcentration, elevated creatinine or lactate, hypoxaemia, pleural effusion, altered mental status, advanced age, obesity, and significant cardiopulmonary or renal comorbidity [21,22,23,24]. The BISAP score is simple and rapidly available, whereas APACHE II and SOFA provide a broader assessment of physiological derangement. Recalculation is more informative than a single admission value.

Patients with shock, persistent hypoxaemia, oliguria, acute kidney injury, altered consciousness, significant electrolyte disturbance, increasing lactate, or evolving organ failure require ICU or high-dependency admission. The threshold should be lower when transfer to a specialist pancreatic centre may become necessary. Early multidisciplinary discussion is warranted for extensive necrosis, suspected abdominal compartment syndrome, gastrointestinal or vascular complications, and patients likely to require advanced endoscopy, interventional radiology, or surgery [2,11,12]. The principal bedside tools and their practical roles are summarised in Table 1.

## 6. Early Critical Care Management

### 6.1. Monitoring and Initial Priorities

Initial management should occur in parallel: confirm the diagnosis, identify the likely aetiology, assess organ function, provide analgesia and oxygen when indicated, begin balanced crystalloid resuscitation, and arrange biliary ultrasonography [2,3,4]. Monitoring intensity should match risk. In unstable patients, arterial pressure monitoring, frequent blood-gas analysis, lactate, hourly urine output, and focused cardiac or lung ultrasound may be appropriate. Central venous access is indicated when vasopressors, difficult access, or complex fluid assessment requires it, not as a routine measure in all patients.

Endpoints should be multimodal: mental status, peripheral perfusion, mean arterial pressure, heart rate, dynamic indices of fluid responsiveness when valid, urine output, BUN/creatinine, haematocrit, lactate, oxygenation, and signs of fluid overload. The target is correction of hypoperfusion while avoiding interstitial oedema, respiratory deterioration, and intra-abdominal hypertension.

### 6.2. Fluid Resuscitation and Haemodynamic Support

Balanced crystalloids, particularly lactated Ringer’s solution, are generally preferred to chloride-rich saline. Current evidence supports moderate, individualised, goal-directed fluid administration rather than routine aggressive loading [2,3,13,20]. In the WATERFALL trial, aggressive resuscitation increased fluid overload without reducing progression to moderately severe or severe disease [20]. These findings should not be interpreted as a reason to undertreat shock; patients with clear hypovolaemia still require prompt resuscitation, but repeated boluses should be justified by evidence of fluid responsiveness and stopped when benefit is absent.

A reasonable approach is a cautious initial bolus in patients with hypovolaemia, followed by lower maintenance rates and reassessment within hours. Patients with heart failure, chronic kidney disease, advanced age, obesity, or established ARDS require particularly conservative titration. If hypotension persists after adequate intravascular volume has been restored, norepinephrine is the preferred first-line vasopressor, with management following general shock principles. Echocardiography helps distinguish vasoplegia, hypovolaemia, right ventricular dysfunction, and sepsis-related myocardial depression.

### 6.3. Analgesia and Sedation

Pain control is essential, but there is no evidence that a specific opioid improves pancreatic outcomes. Intravenous opioids can be titrated safely; concerns about clinically relevant sphincter of Oddi spasm should not result in inadequate analgesia. Paracetamol and, in selected patients without renal dysfunction or bleeding risk, non-steroidal anti-inflammatory drugs can reduce opioid exposure. Thoracic epidural analgesia has physiological appeal and has been investigated in severe AP, but evidence is insufficient for routine use; coagulopathy, infection, haemodynamic instability, and the potential need for urgent procedures frequently limit feasibility. In ventilated patients, sedation should be minimised and managed according to standard ICU principles.

### 6.4. Respiratory Failure

Respiratory compromise may result from atelectasis, pleural effusion, pulmonary oedema, abdominal distension, pneumonia, or ARDS. Supplemental oxygen and high-flow nasal oxygen can be used in selected patients, while non-invasive ventilation requires careful monitoring because abdominal distension, vomiting, and rapid deterioration may increase aspiration and failure risk. Invasive ventilation should use lung-protective tidal volumes based on predicted body weight, appropriate positive end-expiratory pressure, plateau-pressure limitation, and prone positioning for severe ARDS when indicated. Fluid restriction after initial stabilisation is often important because capillary leak and intra-abdominal hypertension worsen lung mechanics.

Pleural effusions rarely require drainage unless large, infected, diagnostically uncertain, or causing significant respiratory compromise. Persistent or recurrent left-sided effusion should raise suspicion of pancreatic duct disruption or pancreaticopleural fistula.

### 6.5. Acute Kidney Injury and Renal Replacement Therapy

Acute kidney injury reflects hypoperfusion, systemic inflammation, sepsis, venous congestion, nephrotoxins, and increased intra-abdominal pressure. Prevention requires avoidance of both under-resuscitation and fluid overload, review of contrast and nephrotoxic exposure, haemodynamic optimisation, and treatment of infection or abdominal compartment syndrome. Renal replacement therapy is initiated for conventional indications such as refractory hyperkalaemia, severe acidosis, fluid overload, uraemic complications, or persistent oliguria with uncontrolled metabolic consequences. There is no established role for routine early haemofiltration solely to remove inflammatory mediators.

### 6.6. Intra-Abdominal Hypertension and Abdominal Compartment Syndrome

Severe AP is a high-risk condition for intra-abdominal hypertension because of visceral oedema, ileus, ascites, fluid loading, and retroperitoneal inflammation. Clinical examination is insensitive; bladder pressure measurement should be considered in patients with tense abdominal distension, worsening ventilation, oliguria, increasing vasopressor requirement, or unexplained organ dysfunction [25]. Intra-abdominal hypertension is defined as sustained or repeated intra-abdominal pressure of at least 12 mmHg, and abdominal compartment syndrome as pressure above 20 mmHg associated with new organ dysfunction.

Management is staged: optimise analgesia and abdominal wall compliance; evacuate gastric and colonic contents; avoid unnecessary positive fluid balance; use diuretics or renal replacement therapy after haemodynamic stabilisation when appropriate; drain significant ascites or collections; and obtain urgent surgical input for refractory abdominal compartment syndrome. Decompressive laparotomy is a rescue intervention, not a routine treatment for pancreatic necrosis. The main intensive care priorities and corresponding treatment goals are summarised in Table 2.

## 7. Nutritional and Metabolic Management

Early feeding is a therapeutic component of care, not merely nutritional support. In mild AP, oral feeding can begin as soon as tolerated without waiting for complete normalisation of pancreatic enzymes. A low-fat solid diet is acceptable and may provide more calories than a stepwise clear-liquid regimen. In moderately severe or severe disease, enteral nutrition is preferred when oral intake is inadequate, because it supports gut barrier function and reduces infectious complications compared with parenteral nutrition [14,26,27].

The PYTHON trial found no advantage of mandatory nasoenteric feeding within 24 h over an oral diet at 72 h with tube feeding on demand in high-risk patients [26]. Thus, early oral intake should be attempted when safe; tube feeding should not be delayed when oral intake is clearly impossible. Nasogastric feeding is usually effective and easier to establish than nasojejunal feeding. Post-pyloric feeding is reasonable with gastric outlet obstruction, severe gastroparesis, recurrent aspiration, or persistent gastric intolerance. Parenteral nutrition is reserved for patients in whom enteral delivery is contraindicated or fails to meet requirements despite optimisation.

Protein targets should reflect critical illness and catabolism, while avoiding overfeeding during the acute phase. Hyperglycaemia, hypocalcaemia, hypomagnesaemia, hypertriglyceridaemia, and refeeding risk require active management. Routine probiotic administration is not recommended; the PROPATRIA trial demonstrated harm in predicted severe AP [28].

## 8. Aetiology-Directed Management

Determining aetiology is essential because recurrence prevention begins during the index admission. Transabdominal ultrasound should be performed to assess gallstones and biliary dilatation. In presumed idiopathic disease, repeat ultrasound, magnetic resonance cholangiopancreatography, or endoscopic ultrasound may reveal microlithiasis, sludge, neoplasia, or structural disease [2,3,4]. Triglycerides, calcium, alcohol exposure, medications, trauma, recent ERCP, and autoimmune features should be assessed.

Urgent ERCP is indicated when biliary AP is accompanied by cholangitis. Persistent biliary obstruction without cholangitis may also justify early intervention after multidisciplinary assessment. In predicted severe biliary AP without cholangitis, routine urgent ERCP did not improve major outcomes in the APEC trial [29]. For mild biliary AP, same-admission laparoscopic cholecystectomy reduces recurrent gallstone-related events and is standard care [30]. In severe disease or substantial collections, cholecystectomy is generally delayed until inflammation and collections stabilise.

Hypertriglyceridaemic AP requires rapid triglyceride reduction through fasting followed by appropriate nutrition, insulin when hyperglycaemia or diabetes is present, and treatment of secondary causes. Plasma exchange can rapidly lower triglycerides but has not shown consistent outcome benefit and should be reserved for selected severe cases, especially when levels remain extreme despite medical therapy or when pregnancy-specific considerations apply.

## 9. Infection, Antibiotics, and Antimicrobial Stewardship

Sterile pancreatic necrosis does not require prophylactic antibiotics. Antibiotics are indicated for extrapancreatic infection, culture-proven infected necrosis, or strong clinical suspicion based on gas within a collection, bacteraemia, sepsis, or otherwise unexplained deterioration [2,3,4,5,31]. Fine-needle aspiration is no longer routinely required when clinical and imaging evidence is persuasive [32]. Blood cultures and cultures obtained during drainage should guide de-escalation.

Agents should have activity against expected enteric Gram-negative and Gram-positive organisms and adequate tissue penetration. Local resistance patterns and prior antimicrobial exposure are important. Routine antifungal prophylaxis is not recommended. The PROCAP trial showed that a procalcitonin-guided algorithm reduced antibiotic prescribing without increasing infection or harm, supporting biomarker-assisted stewardship when interpreted within the clinical context [33].

Persistent fever alone is not proof of infected necrosis. Drug fever, thrombosis, line infection, pneumonia, urinary infection, cholangitis, acalculous cholecystitis, and continuing sterile inflammation should be considered. Conversely, immunosuppressed or elderly patients may develop infection without marked inflammatory signs.

## 10. Necrotising Pancreatitis and the Step-Up Strategy

Most sterile necrosis can be managed conservatively. Intervention is indicated for infected necrosis with ongoing sepsis or clinical deterioration, and for selected sterile collections causing persistent gastric or biliary obstruction, pain, nutritional failure, fistula, or other complications. When the patient can be stabilised, intervention is preferably delayed until necrosis becomes encapsulated, usually around four weeks, because delayed procedures are technically safer and more effective [2,31,34].

The PANTER trial established that a step-up strategy beginning with drainage and escalating to minimally invasive necrosectomy reduced major complications compared with primary open necrosectomy [35]. Endoscopic transluminal drainage is attractive for centrally located collections adjacent to the stomach or duodenum, whereas percutaneous drainage is useful for lateral, paracolic, pelvic, or immature collections and in patients too unstable for endoscopy. Combined endoscopic and percutaneous approaches are often complementary rather than competing.

The PENGUIN trial demonstrated less physiological stress with endoscopic transgastric necrosectomy than surgical necrosectomy [36]. In TENSION, the endoscopic step-up approach did not significantly reduce the composite primary outcome compared with surgical step-up but reduced pancreatic fistula and length of stay [37]. The MISER trial also favoured endoscopic treatment for major complications, costs, and quality of life [38]. Long-term follow-up supports lower rates of pancreatic fistula and reintervention with endoscopic strategies in anatomically suitable patients [39,40].

Timing remains as important as modality. In the POINTER trial, immediate drainage for infected necrosis was not superior to postponed drainage and resulted in more interventions; 39% of patients assigned to postponed intervention were treated without drainage [41]. Thus, antibiotics and intensive support can create time for encapsulation when sepsis is controlled. The DESTIN trial suggested that upfront endoscopic necrosectomy after transluminal access may reduce the number of reinterventions in selected patients, but it does not eliminate the need for individualised assessment of collection maturity, solid burden, bleeding risk, and local expertise [42].

Open surgery retains a rescue role for uncontrolled haemorrhage not amenable to embolisation, bowel ischaemia or perforation, refractory abdominal compartment syndrome, inaccessible necrosis, or failure of less invasive strategies. These decisions should be made in a high-volume multidisciplinary team. The selection and timing of interventions for pancreatic necrosis are summarised in Table 3.

## 11. Special Populations

Obesity and metabolic syndrome increase the probability of severe disease and complicate airway management, ventilation, vascular access, imaging, mobilisation, and fluid assessment [43,44]. Actual body weight should not be used uncritically for all fluid or ventilatory calculations; tidal volume must be based on predicted body weight, and fluid boluses should be driven by physiology rather than weight alone. Visceral adipose tissue may amplify lipotoxicity and systemic inflammation.

Acute pancreatitis in pregnancy and the early postpartum period requires simultaneous maternal stabilisation, fetal assessment when viable, and coordination among intensive care, obstetrics, anaesthesia, gastroenterology, surgery, and neonatology. Gallstones and hypertriglyceridaemia predominate. The maternal indications for organ support, ERCP, and source control remain paramount, while radiation exposure, positioning, aspiration risk, and pregnancy-specific physiology modify technique [45].

Older and multimorbid patients may have less physiological reserve, atypical symptoms, and greater vulnerability to fluid overload, delirium, and deconditioning. Frailty and pre-illness function should inform proportionality of intervention, rehabilitation planning, and shared decision-making without withholding effective organ support solely because of chronological age.

## 12. Emerging Concepts and Future Perspectives

Risk prediction is moving from static admission scores towards dynamic models incorporating repeated vital signs, laboratory trajectories, imaging features, and treatment response. Machine-learning approaches may improve discrimination, but external validation, calibration, explainability, and integration into clinical workflows are essential before routine adoption. Prediction tools should trigger timely review rather than replace expert judgement.

Biomarker research aims to distinguish sterile inflammation from infection and identify patients likely to develop persistent organ failure. Procalcitonin already has pragmatic value for antimicrobial stewardship, while cytokine panels, cell-free DNA, immune phenotyping, and metabolomic signatures remain investigational. A major unmet need is a validated, rapidly available biomarker that changes management rather than merely correlating with severity.

Personalised haemodynamic care is another priority. Future trials should enrol patients with established severe disease, cardiac or renal comorbidity, and distinct shock phenotypes rather than extrapolating from predominantly mild cohorts. Integration of focused echocardiography, venous congestion assessment, lung ultrasound, microcirculatory measures, and intra-abdominal pressure could reduce both occult hypoperfusion and fluid overload.

Interventional care will continue to evolve through improved lumen-apposing stents, dedicated necrosectomy devices, hybrid radiological–endoscopic pathways, and better selection for upfront versus step-up necrosectomy. The central organisational innovation, however, may be regionalisation: patients with complex necrotising disease benefit from early consultation and transfer to centres with advanced endoscopy, interventional radiology, pancreatic surgery, nutrition, and critical care available within a coordinated pathway.

## 13. Conclusions

Severe acute pancreatitis is a dynamic multisystem critical illness in which persistent organ failure, infected necrosis, and iatrogenic complications determine outcome. Modern management is characterised by repeated risk assessment, moderate goal-directed fluid resuscitation, early oral or enteral nutrition, evidence-based organ support, avoidance of prophylactic antibiotics, and delayed minimally invasive source control whenever clinically feasible. The step-up approach has replaced primary open necrosectomy for most patients, while the choice between endoscopic and percutaneous routes should be guided by anatomy, physiology, timing, and local expertise.

**Figure 1:**
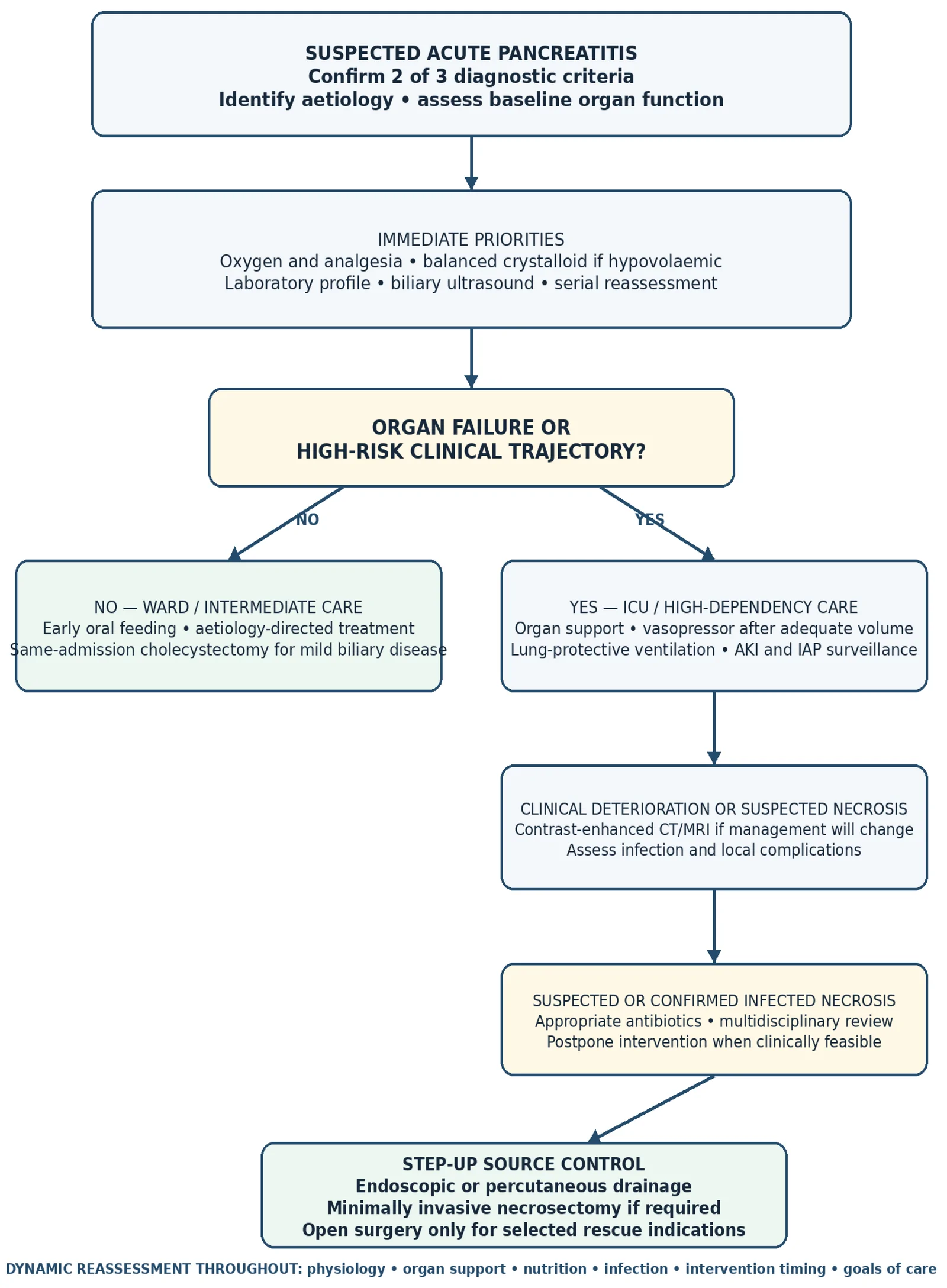
Practical critical care pathway for acute pancreatitis. The pathway is intentionally simplified and must be adapted to patient physiology, collection anatomy, and local expertise. BUN, blood urea nitrogen; IAP, intra-abdominal pressure; ICU, intensive care unit.

The intensivist plays a central coordinating role: preventing fluid overload, recognising respiratory and renal deterioration, monitoring intra-abdominal pressure, supporting antimicrobial stewardship, and ensuring timely multidisciplinary intervention. Future advances are likely to arise from dynamic risk models, phenotype-directed therapy, and regionalised care rather than from a single disease-specific drug. A simplified practical pathway integrating triage, organ support, reassessment, and step-up source control is presented in Figure 1.

## Figures and Tables

**Table 1:** Practical tools for early risk assessment and monitoring.

Tool/Variable	Strength	Limitation	Practical Role
Repeated clinical assessment	Captures trajectory and response to treatment	Requires experienced review and clear escalation triggers	Core method; reassess at least every 6–12 h during the early phase
SIRS	Simple and sensitive for inflammatory deterioration	Low specificity for infection	Persistent SIRS supports higher-acuity monitoring
BUN/creatinine/urine output	Reflects perfusion and renal dysfunction	Affected by baseline renal disease and catabolism	Use trends to guide fluids and identify AKI
BISAP	Rapid five-variable score	Moderate discrimination; should not be used alone	Early communication and triage support
APACHE II/SOFA	Quantifies global physiological and organ dysfunction	More complex; treatment can alter score	Useful in ICU and for serial reassessment
Modified Marshall score	Defines organ failure in the Atlanta classification	Covers only three organ systems	Determines severe AP when failure persists > 48 h
CT Severity Index/modified CTSI	Defines morphology, necrosis, and complications	Early CT may underestimate necrosis	Use when deterioration or intervention planning justifies imaging

**Table 2:** Intensive care priorities in severe acute pancreatitis.

Domain	Preferred Strategy	Avoid	Escalation Trigger
Circulation	Balanced crystalloid; moderate goal-directed dosing; early norepinephrine after adequate volume	Fixed aggressive fluid protocols; persistent positive balance without benefit	Shock, rising lactate, poor perfusion, fluid non-responsiveness
Respiration	Early oxygen assessment; lung-protective ventilation when intubated; prone positioning for severe ARDS	Delayed intubation in failing non-invasive support; excessive tidal volume	Increasing work of breathing, refractory hypoxaemia, altered consciousness
Kidney	Perfusion optimisation; avoid nephrotoxins; monitor urine output and creatinine	Fluid loading solely to force urine output	Refractory electrolyte/acid–base disturbance, fluid overload, uraemia
Nutrition	Early oral feeding when tolerated; enteral tube feeding if oral intake fails	Prolonged fasting; routine parenteral nutrition	Persistent intolerance, ileus, obstruction, severe catabolism
Infection	Culture-guided therapy; antibiotics for proven or strongly suspected infection; consider procalcitonin algorithm	Prophylactic antibiotics for sterile necrosis	Sepsis, gas in collection, bacteraemia, clinical deterioration
Intra-abdominal pressure	Bladder-pressure monitoring in high-risk patients; staged decompression	Ignoring unexplained oliguria or worsening ventilation	IAP > 20 mmHg with new organ dysfunction
Intervention	Multidisciplinary delayed step-up approach when clinically feasible	Early open necrosectomy	Uncontrolled sepsis, bleeding, bowel ischaemia/perforation, refractory ACS

**Table 3:** Selection and timing of interventions for pancreatic necrosis.

Clinical Situation	Preferred Initial Action	Timing	Comments
Sterile necrosis without symptoms	Conservative treatment	No intervention	Observe clinically; repeat imaging only when it will change management
Suspected infected necrosis; stable after antibiotics	Supportive care and multidisciplinary review	Delay towards walled-off stage when feasible	POINTER supports postponement and potential avoidance of drainage
Central walled-off necrosis adjacent to stomach/duodenum	EUS-guided transluminal drainage	Usually ≥ 4 weeks	Escalate to endoscopic necrosectomy if inadequate response
Lateral/paracolic/pelvic collection or early unstable patient	Percutaneous catheter drainage	When source control is required	Can bridge to later endoscopic or surgical treatment
Persistent sepsis after drainage	Endoscopic or minimally invasive necrosectomy	Step-up after reassessment	Approach depends on anatomy and expertise
Bleeding pseudoaneurysm	CT angiography and embolisation	Urgent	Surgery if endovascular control fails
Bowel ischaemia/perforation, refractory ACS, failed minimally invasive care	Surgical rescue	Urgent/individualised	Open necrosectomy is no longer first-line but remains essential in selected cases

## Data Availability

No new data were created or analyzed in this study. Data sharing is not applicable to this article.
